# On the public dissemination and open sourcing of ultrasound resources, datasets and deep learning models

**DOI:** 10.1038/s41746-025-02162-4

**Published:** 2025-11-24

**Authors:** Mohammad Alsharid, Xiaoqing Guo, Qianhui Men, Pramit Saha, Divyanshu Mishra, Rahul Ahuja, Cheng Ouyang, J. Alison Noble

**Affiliations:** 1 Institute of Biomedical Engineering, Department of Engineering Science, Roosevelt Drive, Old Road Campus Research Building, Headington, Oxford, Oxfordshire United Kingdom; 2 Department of Computer Science, Khalifa University, Shakhbout Bin Sultan Street, Hadbat Al Za’faranah, Zone 1, Abu Dhabi, Abu Dhabi United Arab Emirates

**Keywords:** Medical imaging, Ultrasonography, Computer science

## Abstract

Ultrasound data is relatively under-utilized in machine learning applied to medical imaging research when compared to other imaging modalities. Towards rectifying this, this paper (and the associated webpage) catalogs and assesses the usability of publicly available ultrasound datasets and models. Datasets were categorized and ranked using an original dataset quality score, SonoDQS. The models were scored using our model quality score, SonoMQS. We identified 72 public ultrasound datasets covering different anatomies and collected in different parts of the world. We identified 56 open-source models trained on ultrasound data. Most open-source models were trained on datasets that are or were made publicly available. A plurality of the datasets are of similar quality, corresponding to bronze (fifth tier) in the SonoDQS ranking. There are a few publicly available datasets of fetal content (5) and prostate anatomy (4) in spite of the wide use of ultrasound in these clinical areas, acknowledging a notable gap.

## Introduction

Ultrasound is relatively underrepresented in research in the machine learning applied to medical imaging space when compared to other modalities such as magnetic resonance imaging (MRI) and X-ray computer tomography (CT). By way of illustration, at a leading international conference in medical image analysis in 2023 (Medical Image Computing and Computer-Assisted Interventions, MICCAI 2023)^1^, only 51 papers were classed under the ultrasound modality topic. On the other hand, there were 185 papers and 126 papers that were classed under the MRI and CT topics, respectively. This observation holds true for earlier years as well. In MICCAI 2022, the equivalent figures are 43 ultrasound, 159 MRI and 82 CT papers^2^ and in MICCAI 2021, the figures are 32 ultrasound, 128 MRI, and 95 CT papers^3^. This holds despite the wide variety of tasks performed on ultrasound imaging data, including image classification^4–6^, image captioning^7–10^, video captioning^11,12^, representation learning^13–15^, and segmentation^16,17^.

In this paper, we report on a study to collate, analyze and score publicly available ultrasound datasets and models. Our goal was to identify open-source resources available for ultrasound imaging research, including machine learning-based analysis and sonography data science, to encourage the scientific community to answer more research questions pertaining to clinical ultrasound.

There are several related reviews on public datasets for other medical imaging modalities and clinical imaging areas. Khan et al.^18^ reviews publicly available ophthalmological imaging datasets and scores them based on how much information on them is made available. Magudia et al.^19^ discuss the challenges of assembling large medical datasets and share best practices on cohort selection, data retrieval, storage, and failure handling. Kohli et al.^20^ highlight the need for improved collection, annotation, and reuse of medical imaging data, as well as the development of best practices and standards. These works^19,20^ suggest there is a need to standardize how datasets are described and what information to track when they are collected and prepared. In this paper, we describe the methods used in our search for public ultrasound datasets and open-source models and detail what we have found available and any interesting points to note relating to the public resources.

To build AI-powered clinical solutions that generalize well, researchers need access to datasets on which to train their proposed models on. Ideally, they would train on a variety of datasets that have different characteristics, such as the country of origin, the type of scanning device used, and the time-frame during which the data was collected.

AI-powered clinical solutions that could be of benefit to current medical practice can vary. One way, for example, would be through solutions that could allow for assistive tools that speed up the scanning process (e.g., automatic segmentation of anatomies of interest), leading to direct benefits to clinician productivity, allowing medical facilities to serve more people in the same window of time.

## Results

### Findings of the dataset search

**Fig. 1:**
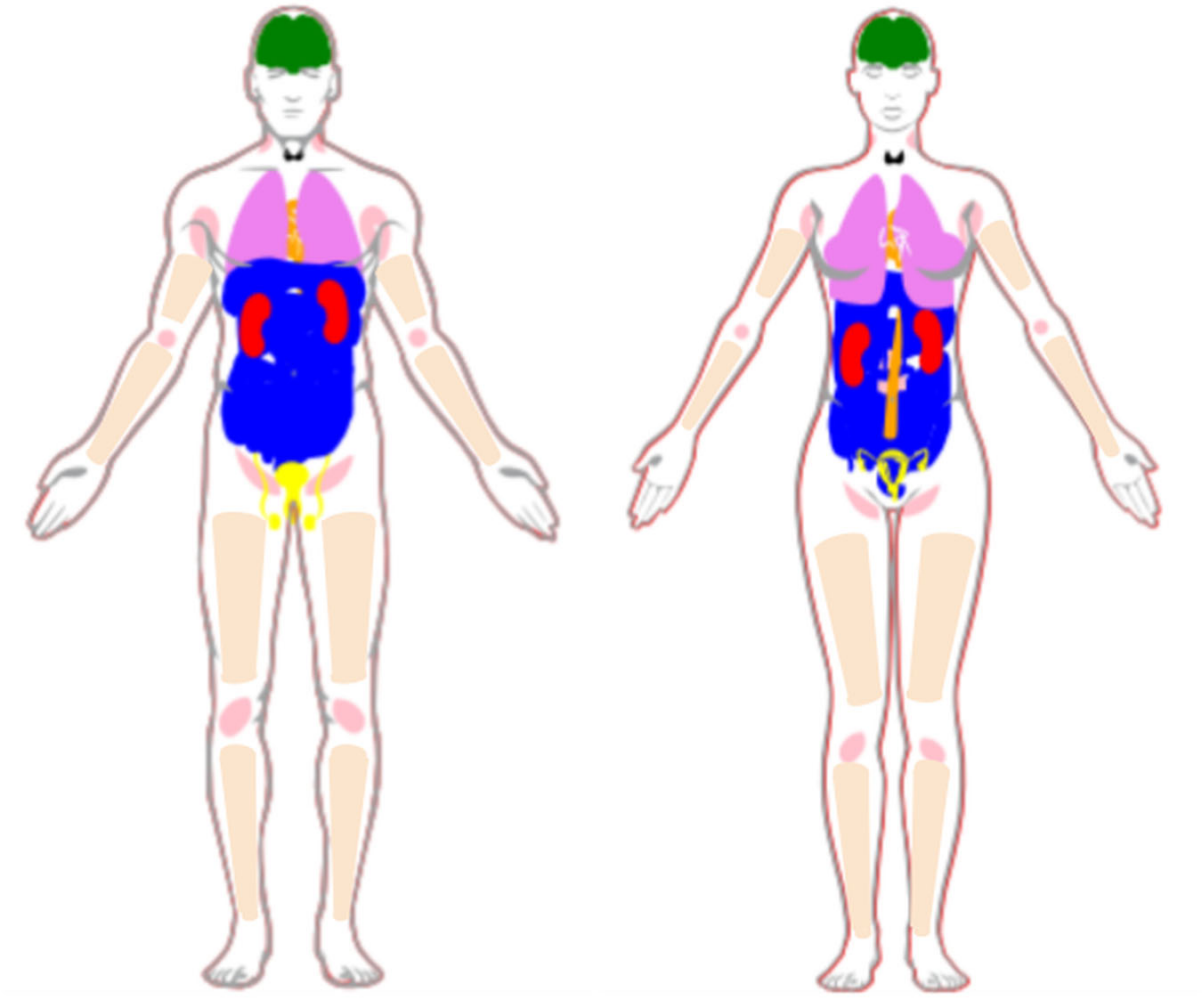
Human anatomy diagrams showing what anatomy groups are represented in the publicly available US datasets. Left shows male anatomy, right female anatomy. The colors orange, green, black, blue, red, violet, pink, yellow, and peach represent parts of the circulatory system, the brain, the thyroid gland, parts of the digestive system, the kidneys, the lungs and the breasts, lymph nodes, the reproductive system, and the musculoskeletal system, respectively. The fetus is not shown in the anatomy diagrams. Plot was made using the pyanatomogram library^180^. Anatomogram silhouettes © EMBL-EBI Expression Atlas, CC BY 4.0^181^.

**Fig. 2:**
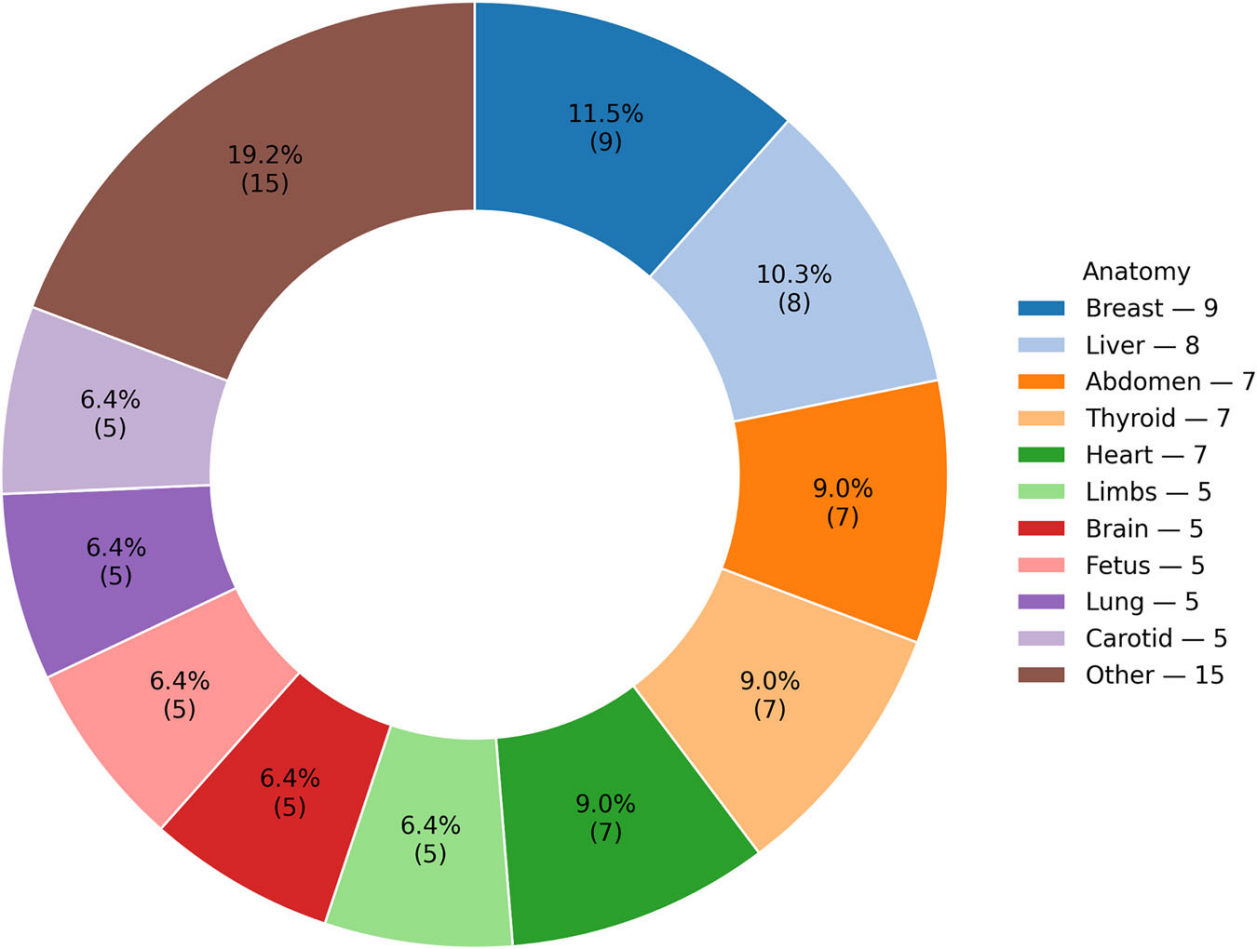
Pie chart showing the breakdown of different anatomy groups in the publicly available US datasets. The numbers shown are the number of datasets that contain data samples of this anatomy group. Some datasets contain multiple anatomy groups. The plot was made using the python library Matplotlib^182^.

**Fig. 3:**
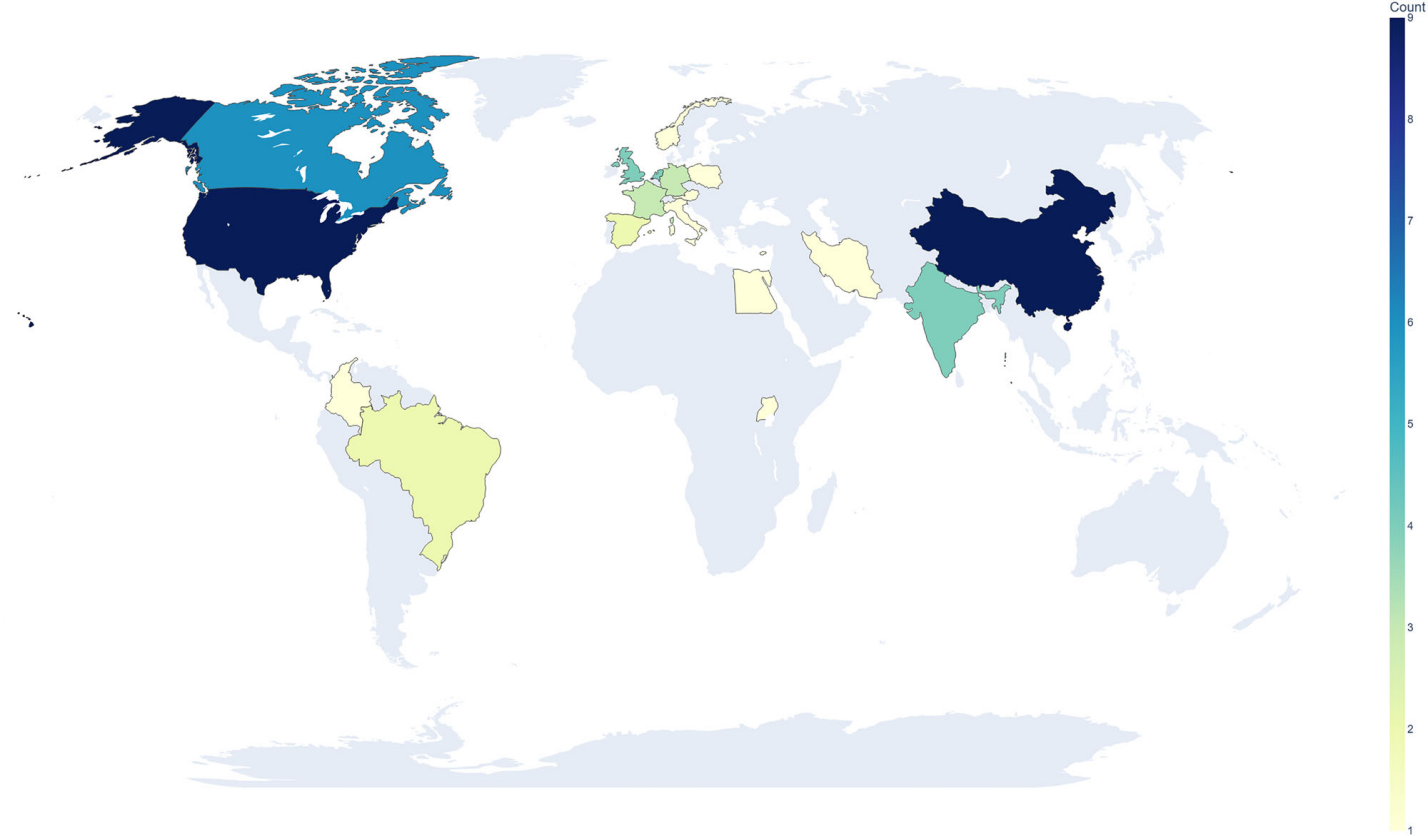
The geographical distribution showing where the reported datasets were acquired is shown. Note that datasets that do not mention where the data is acquired are not included. This map, plotted using the python library plotly^183^, is provided for illustrative purposes only and does not reflect any legal or political stances. Basemap: Natural Earth (Public Domain).

**Fig. 4:**
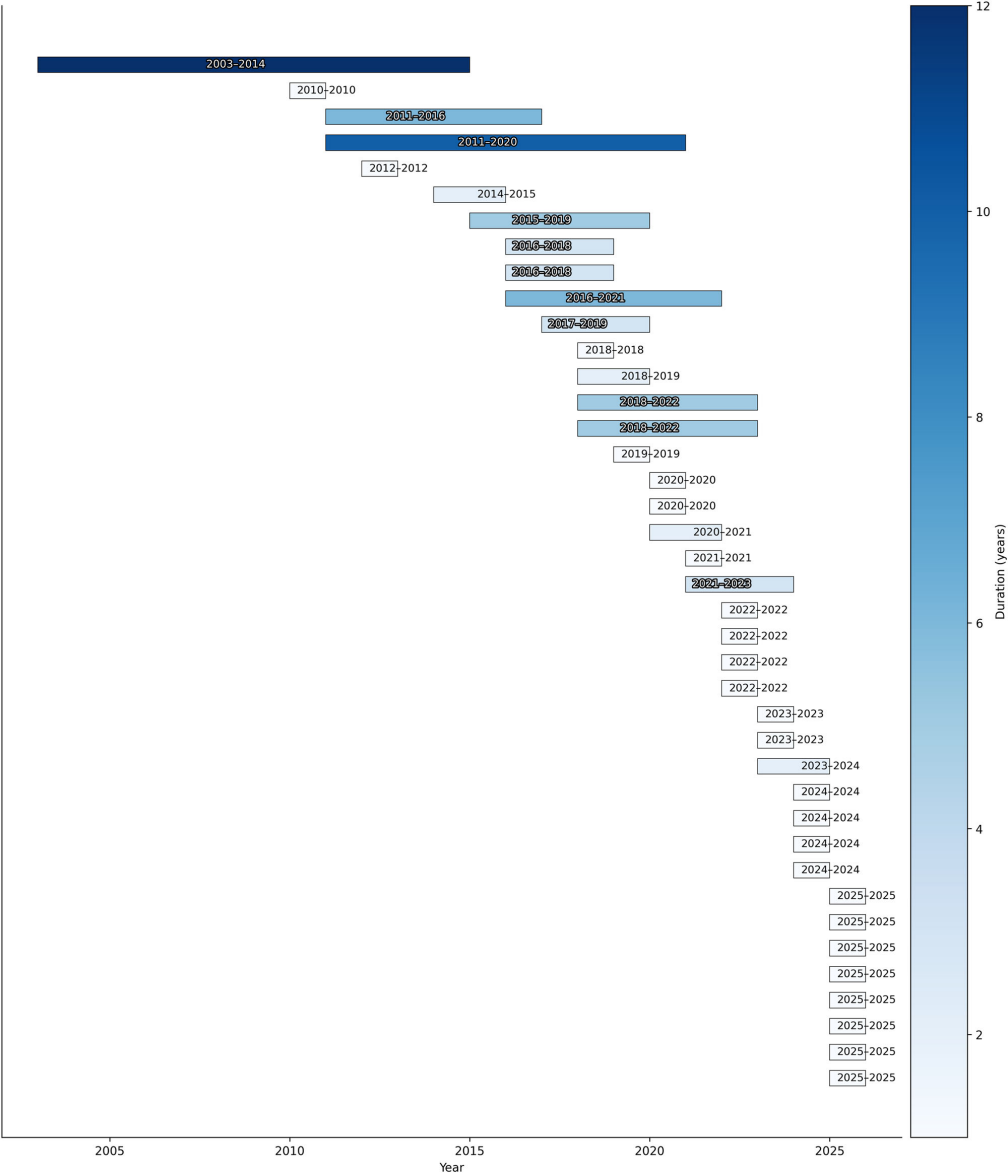
A chart showing the ranges of acquisition in years of different datasets. Please note this only includes datasets that mention ranges of acquisition. The plot was made using the python library Matplotlib^182^.

The results of the dataset search suggest that there are a number of different anatomies represented in open-source ultrasound datasets, as shown in Figs. 1 and 2. It is interesting to note from Table 1 that our search implies that the popularity of using ultrasound imaging in clinical practice or clinical research does not translate to the availability of public datasets. An example is the prostate, where only four publicly available datasets were identified. Fetus and fetal related anatomies are among the less prevalent anatomies in public US datasets, as shown in Fig. 2. In Figs. 3 and 4, we summarize where and when publicly available US datasets are being acquired and collected, respectively. These are but two ways to visualize some of the characteristics of the datasets.

**Table 1:** Summarized characteristics of the open-sourced ultrasound datasets

Dataset name	Anatomy	Total dataset size	Open access (OA) or available on request (AR)	SonoDQS
FETAL PLANES DB^34^	Fetus, fetal abdomen, fetal brain, fetal femur, fetal thorax, maternal cervix	12,400	OA	Gold
RESECT^35^	Brain	23	OA	Gold
DDTI^36^	Thyroid	481	OA	Silver
Ultrasound nerve segmentation^37^	Brachial plexus	16,778	OA	Bronze
Ultrasound image enhancement^38^	Thyroid, liver, breast, kidney, and carotid artery	2464	OA	Bronze
Tufts medical echocardiogram dataset (TMED)^39^	Aortic stenosis, heart	6567	AR	Bronze
COVIDx-US^40^	Lung	242	OA	Bronze
The open kidney ultrasound data set^41^	Kidney	514	AR	Gold
FPUS23^42^	Ultrasound fetus phantom	15728	OA	Steel
Thyroid ultrasound cine-clip^43^	Thyroid	167	AR	Steel
Reconstructing of 2D ultrasound (US) images into a 3D volume	Forearms	2400	AR	Bronze
Fetal abdominal structures segmentation dataset using ultrasonic images	Fetal abdomen, fetal liver, fetal stomach, fetal aorta artery, fetal spine, intrahepatic portion of the umbilical vein	1588	OA	Gold
Echocardiogram videos (EchoNet-Dynamic)^44,45^	Heart	10300	AR	Silver
FALLMUD: Fascicle lower leg muscle ultrasound dataset^46,47^	Lower leg muscles	812	OA	Steel
Micro-ultrasound prostate segmentation dataset^48^	Prostate	75	OA	Bronze
CAMUS^49,50^	Heart	500	OA	Silver
Echocardiography (EchoNet-LVH)^51,52^	Heart	12000	OA	Silver
Regensburg Pediatric Appendicitis Dataset^53,54^	Pediatric appendicitis, appendix, abdomen’s right lower quadrant, intestines, lymph nodes and reproductive organs.	2097	OA	Bronze
Gallbladder ultrasound video^55,56^	Gallbladder	64	AR	Bronze
Gallbladder cancer ultrasound^57,58^	Abdomen, gallbladder	1255	AR	Silver
Breast lesion detection in ultrasound videos (BUSV)^59,60^	Breast	188	OA	Silver
Muscle-tendon junction tracking in ultrasound images^61–64^	Muscle-tendon junction (MJT)	1344	AR	Bronze
Breast ultrasound images dataset^65,66^	Breast	780	OA	Bronze
BITE: Brain images of tumors for evaluation database^67,68^	Brain	14	OA	Silver
Dermatologic ultrasound images for classification^69,70^	Skin	202	OA	Steel
Polycystic ovary ultrasound images dataset^71^	Ovary	54	OA	Bronze
CUBS^72^	Carotid	2176	OA	Gold
Knee ultrasound dataset in a population-based cohort^73^	Knee	7571	OA	Silver
BioBank: Carotid artery ultrasound image (left) (20222) and (right) (20223)^74^	Carotid artery	43195	AR	Steel
ReMIND2Reg^75^	Brain	104	OA	Silver
ReMIND^76^	Brain	114	OA	Silver
HC18^77,78^	Fetal head	1334	OA	Bronze
KFGNet^79^	Thyroid	3668	OA	Bronze
BUSIS^80^	Breast	562	OA	Steel
Ultrasound Elastography^81–84^	Liver cancer	2000	OA	Steel
TUS-REC^85^	Arm, forearm	1200	OA	Steel
Breast ultrasound B dataset^86,87^	Breast	163	OA	Bronze
Thyroid^88,89^	Thyroid	214	OA	Bronze
CCA^90,91^	Carotid	2307	OA	Bronze
Chinese ultrasound-report dataset^92,93^	Breast, thyroid, and liver	7390	OA	Bronze
GDPH & SYSUCC^94^	Breast	2405	OA	Bronze
STU-Hospital^95^	Breast		OA	Steel
mu-Reg^96^	Prostate		OA	Steel
mu-RegPro^97,98^	Prostate	108	OA	Steel
UCLA^99^	Prostate	1151	OA	Steel
LEPset^100^	Pancreas	11500	OA	Steel
Geometric ultrasound localization microscopy^101,102^	Microbubbles in the vasculature		OA	Steel
BioBank carotid (30005)^103^	Carotid artery	83193	AR	Steel
COVID-BLUES^104^	Lung	362	OA	Platinum
POCUS Dataset for COVID-19 Detection^105^	Lung	211	OA	Bronze
MIMIC-IV-ECHO^106^	Heart	525,000	AR	Gold
Ultrasound guided regional anesthesia^107,108^	Brachial plexus nerves	277	OA	Platinum
Unity Imaging Colloborative^109^	Heart	75000	OA	Bronze
C-TRUS Dataset^110,111^	Abdomen, transabdominal, colon wall	827	OA	Steel
ACOUSLIC-AI^112^	Fetal Abdomen	600	OA	Platinum
PCOSGen^113,114^	Ovary	4668	OA	Bronze
B-mode-and-CEUS-Liver^115^	Liver	120	AR	Bronze
BEHSOF^116^	liver	113	OA	Bronze
LUMINOUS^117^	Lumbar multifidus muscle	341	OA	Bronze
liver_ultrasound computer vision project^118^	Liver	400	OA	Bronze
Dataset of B-mode fatty liver ultrasound images^119^	Liver	550	OA	Diamond
Annotated ultrasound liver images dataset^120^	Liver	735	AR	Bronze
TED project^121^	Heart	98	OA	Bronze
BUS-BRA^122,123^	Breast	1875	OA	Gold
TN5000^124^	Thyroid	5000	OA	Platinum
Cardiac assessment and classification of ultrasound (CACTUS)^125^	Heart phantom	37,736	OA	Gold
POCUS LUS datasets^126^	Lung	10	OA	Bronze
High-resolution ultrasound data for AI-based segmentation in mouse brain tumor^127,128^	Non-human	1856	OA	Platinum
Dataset of 3D ultrasound neuroimages^129^	Brain	5	OA	Bronze
A dataset of lung ultrasound images for automated AI-based lung disease classification^130^	Lung	1062	OA	Silver
Ultrasound normal kidney image computer vision dataset^131^	Kidney	1080	OA	Steel
FOCUS: Four-chamber ultrasound image dataset for fetal cardiac biometric measurement^132^	Fetal cardiac	300	OA	Silver

The full table can be found on the webpage.

**Fig. 5:**
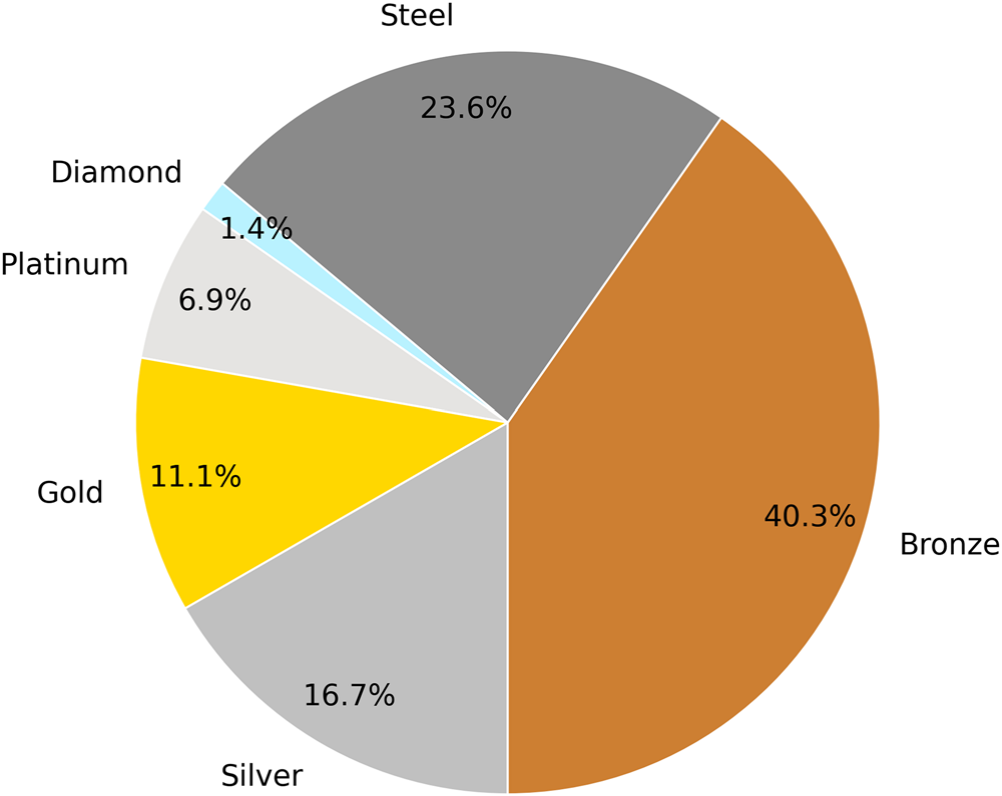
A pie chart showing the classification of datasets according to the SonoDQS levels as a percentage of the total number of datasets. The plot was made using the python library Matplotlib^182^.

**Fig. 6:**
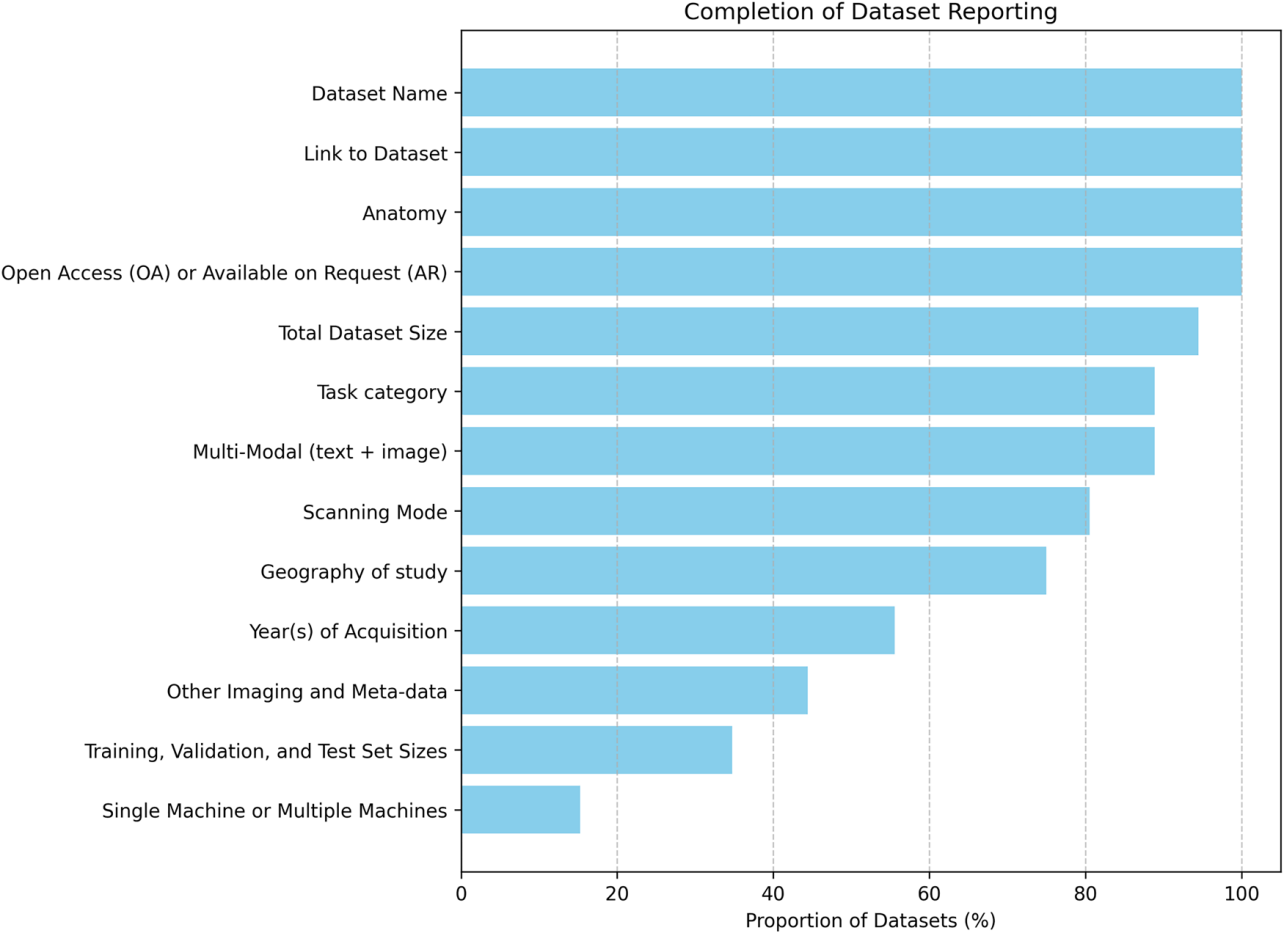
A bar chart showing the completeness of the datasets in terms of the 13 most commonly shared characteristics. 100% means this characteristic or information is always shared with datasets. The plot was made using the python library Matplotlib^182^.

In our proposed SonoDQS dataset quality score, there are seven possible tiers or ranks: diamond, platinum, gold, silver, bronze, steel, and unrated. A plurality (40.32% of the datasets are of similar quality, corresponding to bronze (fifth tier out of seven tiers) in the SonoDQS ranking. The classification of the datasets according to the SonoDQS ranks can be found in the pie chart in Fig. 5. Some dataset characteristics are considered to be more important when showing how complete a dataset is Fig. 6 shows these characteristics, and it is pleasing to note that most of these characteristics are shared and made available with their corresponding datasets.

## Discussion

We identified 72 public ultrasound datasets covering different anatomies and coming from different parts of the world. We observed that anatomies such as fetal content and prostate did not have many public datasets as one might have expected given the wide spread use of ultrasound in these clinical ultrasound areas. We identified 56 open-source models trained on ultrasound data. It is noteworthy that when open-sourcing models, researchers share more comprehensive information about the models than they do when sharing the dataset.

Most open-source models 30/56 actually do not publicly make available their model weights; however, they still met our definition of open source because the code, the dataset, and the training details are available.

We recognize that the boundary thresholds set for SonoDQS ranks were set empirically and could undergo refinement in the future. The ranks, nonetheless, are useful as a guide to help in the selection of the datasets to consider and a way to look at the quality of ultrasound datasets as whole. A researcher is likely to use the dataset characteristics when deciding which exact datasets are useful for their purpose. The fact that there are few open-sourced fetal ultrasound datasets and prostate ultrasound datasets is particularly noteworthy. The published literature in this area is primarily on private data.

The proposed SonoDQS and SonoMQS scores fundamentally are meant to encourage the consistent sharing of comprehensive information on public ultrasound datasets and open-source models in a standardized manner. This consistent sharing of relevant comprehensive information in a standardized manner is important to clinical decision making and ultrasound diagnostics. If, for example, a hospital is interested in using a readily-available model but would want one trained on data from their geographic region or trained on data acquired with equipment similar to what they have in-house. Being open about such details ensures greater transparency which is important in the clinical space but also is reassuring to potential users because they know more about the datasets and the models. Potentially, being more open about these details might help slightly with clinical translation.

We acknowledge the existence of limitations with our work. In terms of datasets, we only focus on publicly available datasets disregarding in our analyses and reporting private datasets that are publicly known to exist but are not available. The number of SonoDQS ranks is somewhat subjective (diamond, platinum, etc.) despite going through an iterative process. They exist to make it easy to quickly compare the SonoDQS scores of a large number of datasets.

This paper catalogs open sourced ultrasound datasets and ranks them based on how open they are and how much information has been shared about the data. Open source models are also reported and ranked in terms of how open-sourced they have been made. For the reporting of both the public datasets and the open-sourced models, the cut-off date was September 2025.

## Methods

### Identification of publicly available ultrasound imaging datasets

Team members searched for information on datasets and models starting in April 2024 and ending in September 2025 The search strategies are described next. Each team member was tasked with searching and identifying publicly available ultrasound datasets. This was achieved in a number of ways, such as through search engines (most typically Google search^21^), the website Paperswithcode^22^, WeChat search^23^, social media, blogs, Google Scholar^24^, ChatGPT^25^ and its ability to conduct searches through Bing^26^, survey papers^27^, and Google Dataset^28^. The search terms that were used to identify and locate open-source ultrasound imaging datasets include “ultrasound imaging”, “public ultrasound datasets”, and “ultrasound”.

One important characteristic is dataset access. A dataset may be available immediately as an open access dataset or available on request which may require the signing of an agreement before the dataset can be downloaded. This is similar to what was done by ref. ^18^ for opthalmological datasets who also noted whether publicly available opthalmological datasets were open-access or available on request.

There are several dataset characteristics that we attempted to identify. We based these on the checklist used by organizers of MICCAI 2024 Challenges^29^. This collected information goes into tables, the summary table and the detailed table. The 13 more important dataset characteristics of interest: (1) dataset name, (2) link to dataset, (3) anatomy, (4) scanning mode, (5), multi-modal (text+image), (6) total dataset size, (7) training, validation, and test set sizes, (8) task category, (9) geography of study, (10) year(s) of acquisition, (11) other imaging and metadata, (12) single machine or multiple machines (single center or multiple centers), and (13) open access (OA) or available on request (AR). Please find the interactive table on display on the webpage interface.

There are 20 further characteristics that are relatively less important but are still collected and considered. These characteristics are: (1) device(s) used to acquire data (US machine type and transducer), (2) data acquisition protocols, (3) center or institute collecting or providing data, (4) characteristics of the data acquirers (such as their experience level), (5) other characteristics of the training, validation, and testing datasets (such as the data distribution), (6) what is considered a single data sample, (7) number of operators or sonographers, (8) how annotations are made (manually or automatically) and by how many annotators, (9) the instructions given to the annotators, (10) annotation description, (11) how were annotations merged together if required, (12) dataset application cases (such as education), (13) ethics approval statement, (14) data usage agreement, (15) challenge if dataset is associated with a challenge, (16) imaging modalities and techniques used, (17) context information relevant to the spatial or spatio-temporal data, (18) patients’ (or subjects’) details, such as gender, age, and medical history, (19) Doppler imaging (Y/N), and (20) B-mode imaging (Y/N).

For ease of readability of this paper, a reduced version of the table is included as Table 1. This table has four columns: (1) the name of the dataset, (2) the anatomies involved, (3) the total size of the dataset, and (4) whether the dataset is open access or available on request. The full table webpage has been made uploaded as additional material as HTML files.

Another characteristic of note is the scanning mode. We have specified five possible scanning modes: (1) 2D ultrasound images from freehand scanning potentially containing both standard and non-standard planes for the scanned anatomy, (2) 2D ultrasound images meant solely for diagnostics and measurements typically containing only standard planes for the scanned anatomy, (3) 2D+t content (i.e. ultrasound videos), (4) 3D ultrasound images (there is different physics involved and different probes used compared to 2D imaging), and (5) 3D ultrasound images reconstructed from 2D ultrasound images.

Dataset characteristics were extracted from the source material by the team member that found the dataset during the data search stage. Subsequently, a second team member verified and corrected, if necessary, the characteristics. This cross-referencing was done, as a quality assurance step, to ensure the integrity of the content in the paper, the webpage, and their associated tables.

### Identification of open-source ultrasound imaging models

On 7 July 2024, in a lecture titled “Collaborate as a Community: Innovations and Trends in Open-Source AI”, presented at the Machine Learning for Health & Bio track of the Oxford Machine Learning Summer School (Oxford, UK), Vincent Moens described four elements of a trained model that relate to its “openness”: (1) the code, (2) the training data, (3) the model weights, and (4) the training procedure details (unpublished). A fully open model will provide details on all four elements, and a closed model provides no detail. Models may also have different degrees of openness between these two extremes. While a fully open model is desirable, a partially open model may still be very useful for community research and allow for model comparisons.

Any deep learning models trained on ultrasound data were eligible for inclusion. The search was conducted in two stages. First, we performed a literature search to identify studies related to ultrasound data analysis published since 2022. This search focused on top journals and conferences in medical imaging, including *IEEE Transactions on Medical Imaging*, *Medical Image Analysis*, and the *International Conference on Medical Image Computing and Computer-Assisted Intervention (MICCAI)*. We used keywords describing various types of ultrasound, including ‘ultrasound’, ‘US’, ‘sonography’, and ‘sonographer’. This returned 112 *IEEE Transactions on Medical Imaging* papers, 170 *Medical Image Analysis* papers, and 97 MICCAI papers. In the second stage, we conducted a targeted search on GitHub, using similar keywords to identify highly starred repositories published since 2022. We reviewed the top results from the first ten pages after sorting by stars.

For each identified paper or Github repository, we checked whether open-source code and model weights were provided. If the training data is open source in addition to the training details and the code is shared, then even if model weights are not available, we recorded it as an open-source model. We did this because, theoretically, given the code, the training details, and the training data, one could obtain the weights by training the model the same way on the same data. The search for the two sources was done from August 2024 to December 2024.

The search yielded 52 open-source models that met our criteria. In summary, to find the open-source models, we have perused the Github pages of many publications published as part of the main conferences of MICCAI 2024, MICCAI 2023, and MICCAI 2022 as well as the journals Medical Image Analysis (MedIA) and IEEE Transactions in Medical Imaging (TMI).

From July 2025 to September 2025, we later expanded our search strategy to ensure a broader and more systematic coverage of the literature. Specifically, we used Google Scholar to search for ultrasound deep learning models published after 2022 Year using the keywords “ultrasound” + “deep learning”. We reviewed the first 20 pages of results for each query, which corresponds to several hundred candidate works, and selected representative high-quality papers based on (i) publication in top-tier venues, (ii) relevance to ultrasound modeling, and (iii) shared a link to a GitHub repository. This brought the total number of found open-source models to 56.

There are specific characteristics we report for open-source models, including direct links to the open-source code, the task the model was designed for, the corresponding open-source dataset and its accessibility, training details, availability of model weights, competing interest statements, authorship and contribution statements, and software citations. These characteristics then make up the columns of the model table as shown in Table 2. We categorize the models based on 13 different tasks: Image enhancement, generation, pretraining/foundation model, detection, segmentation, estimation, classification, post-processing, report generation, scanning-guide, explainability, reconstruction, and registration. Here, Image Enhancement implies enhancing the low quality ultrasound images to the high-quality images. Post-processing refers to the matching of clinical post-processing techniques typically found in commercial ultrasound scanners. Estimation means prediction or quantification of different diagnostic parameters from ultrasound images such as ejection fraction, biometry, displacement, etc. Scanning-guide indicates automated scanning guidance algorithm designed to help sonographers capture high-quality ultrasound images.

**Table 2:** Open-sourced ultrasound models and some of their characteristics: the availability of their training dataset, weights, and code, task, and score

Model name	Public training dataset	Public model weights	Shared code	Task	SonoMQS
An Annotation-free Restoration Network^133^	Yes	Yes	Yes	Image Enhancement	0.81
Ultrasound Fetal Brain Imaging Synthesis^134^	Yes	No	Yes	Generation	0.69
USFM^135^	Yes	Yes	Yes	Pretraining / Foundation Model	0.81
USCL^13^	Yes	Yes	Yes	Pretraining / Foundation Model	0.81
Explainable US^136^	Yes	Yes	Yes	Detection	0.81
AAU-net^137^	Yes	No	Yes	Segmentation	0.63
SAMUS^138^	Yes	No	Yes	Segmentation	0.69
EchoNet Dynamics^139^	Yes	No	Yes	Segmentation, Estimation	0.69
EchoNet-LVH^52^	Yes	Yes	Yes	Estimation	0.81
US_UCL^55^	Yes	Yes	Yes	Pretraining/Foundation Model	0.81
FocusMAE^140^	Yes	Yes	Yes	Classification	0.81
Deep MTJ	Yes	Yes	Yes	Estimation	0.81
EchoCLIP^141^	Yes	Yes	Yes	Pretraining/Foundation Model	0.81
Echo from noise^142^	Yes	Yes	Yes	Generation	0.81
KGA-Net^143^	Yes	No	Yes	Classification	0.63
CVA-Net^59^	Yes	Yes	Yes	Detection	0.81
MimickNet^144^	Yes	Yes	Yes	Post-processing	0.81
PLD-AL^145^	Yes	No	Yes	Segmentation	0.56
UltraDet^146^	Yes	Yes	Yes	Detection	0.81
MHVAE^147^	Yes	No	Yes	Generation	0.69
PAD-detection^148^	Yes	No	Yes	Detection	0.50
LOTUS^149^	Yes	No	Yes	Segmentation	0.69
fetal biometry^150^	Yes	No	Yes	Estimation	0.63
KFGNet^151^	Yes	No	Yes	Detection	0.63
EchoGraphs^152^	Yes	No	Yes	Segmentation	0.69
USG-Net^153^	Yes	Yes	Yes	Segmentation	0.81
CACTUSS^154^	Yes	No	Yes	Segmentation	0.69
PICTURE^155^	Yes	Yes	Yes	Scanning-Guide	0.75
Multi-Level-Global-Context-Cross-Consistency^156^	Yes	No	Yes	Segmentation	0.69
CMU-Net^157^	Yes	No	Yes	Estimation	0.67
EchoDiffusion^158^	Yes	No	Yes	Segmentation	0.69
xai_thyroid^159^	No	Yes	Yes	Explainability	0.69
LGRNet^160^	No	Yes	Yes	Segmentation	0.69
NR-Rec-FUS^161^	Yes	No	Yes	Reconstruction	0.69
BU-Mamba^162^	Yes	Yes	Yes	Classification	0.81
CU-Reg^163^	Yes	No	Yes	Registration	0.69
Un-supervised Segmentor 4Ultrasound^164^	Yes	No	Yes	Segmentation	0.69
MI-SegNet^90^	Yes	No	Yes	Segmentation	0.69
echo-stgnet^165^	Yes	No	Yes	Detection	0.75
FUNSR^166^	Yes	No	Yes	Reconstruction	0.88
4D Heart Model^167^	Yes	No	Yes	Generation, Estimation	0.75
semi-supervised-multiview-cbm^54^	Yes	No	Yes	Explainability	0.75
RadFormer^168^	Yes	Yes	Yes	Classification	0.88
IFT-Net^169^	Yes	No	Yes	Estimation	0.75
semi-supervised-shadow-aware-network^170^	Yes	No	Yes	Segmentation	0.69
HoVerTrans^94^	Yes	No	Yes	Classification	0.69
AAU-net^137^	Yes	No	Yes	Segmentation	0.63
Ultrasound-report-generation^171^	Yes	No	Yes	Report Generation	0.63
RF-ULM^172^	Yes	No	Yes	Detection	0.63
DSMT-Net^173^	Yes	No	Yes	Classification	0.63
Meta-USCL^174^	Yes	Yes	Yes	Pretraining/Foundation Model	0.81
BEDLUS^175^	Yes	Yes	Yes	Localization	0.8125
GBC-Net^176^	Yes	Yes	Yes	Detection	0.9375
MicroSegNet^177^	Yes	Yes	Yes	Segmentation	0.9375
FUVAI^178^	No	Yes	Yes	Estimation	0.75
SRML-1D^179^	No	Yes	Yes	Localization	0.5625

### Scoring datasets and models

We draw inspiration from the data quality score used by Health Data Research UK (HDRUK)^30,31^ to define an ultrasound dataset quality score. In this context, datasets are not scored based on the quality of the data samples or how well-annotated they are, but rather on how complete the information shared on the dataset is. We have adapted the HDRUK score, and for the sake of clarity, we refer to our modified HDRUK score as SonoDQS.

It is worth noting that the score components are weighted. Different characteristics that contribute to SonoDQS have different weights associated with them based on their relative importance. For SonoDQS, the weighting of the different characteristics was chosen as follows. The characteristics that we consider more important make up the characteristics that are part of the summary table. These characteristics have been given twice as much weight as those only in the detailed table.

1 $${\text{Weighted}}\,{\text{Completeness}}\,{\text{Percent}}=\sum ({\text{weights}}\,{\text{of}}\,{\text{completed}}\,{\text{fields}})$$

2 $${\text{SonoDQS}}=\frac{{\text{Weighted}}\,{\text{Completeness}}\,{\text{Percent}}}{{\text{Total}}\,{\text{number}}\,{\text{of}}\,{\text{characteristics}}}$$

SonoDQS quantifies the completeness of data entries by weighting completed fields, summed in the numerator. The denominator is the total number of dataset characteristics. The result provides a percentage that reflects the extent of completeness, adjusted for the relative importance or weight of the different characteristics.

HDRUK scores are binned into four levels: “bronze”, “silver”, “gold”, and “platinum”^30^. For SonoDQS we also use “platinum”, “gold”, “silver”, and “steel” as rankings. However, additionally we include “unrated”, which denotes datasets that that have significant gaps in data reporting or unverifiable details.

Initially, for SonoDQS, we assigned a level of “platinum” for scores ≥0.6, “gold” for scores <0.6 and ≥0.5, “silver” for scores <0.5 and ≥0.4, and “bronze” for scores below 0.4. Level boundaries were subsequently refined empirically as follows.

Firstly, to emphasize the greater quality of platinum and gold, we adjusted the level thresholds as follows: ≥0.7 for the “platinum” level, <0.7 and ≥0.55 for “gold”, <0.55 and ≥0.25 for “silver”, <0.25 and ≥0.1 for “bronze”. Datasets that score <0.1 are considered “unrated”.

The thresholds were further refined so that the threshold for silver became ≥0.57. Two more levels: “diamond” (to be the highest) and “steel” (to be above “unrated”) were subsequently introduced. The final levels were fixed at “diamond” ≥0.87, “platinum” <0.875 and ≥0.77, “gold” <0.77 and ≥0.67, “silver” <0.67 and ≥0.57, “bronze” <0.5 and ≥0.45, “steel” <0.45 and ≥0.32, and “unrated” <0.32. The SonoDQS level boundary values were set early while dataset collection was still ongoing (when no more than 32 datasets had been found) to define wide, non-overlapping ranges even as we expanded from four to five to seven levels. These values were agreed on by our internal team, ensuring a coverage of a wide range of values, and then fixed for all subsequent analyses, avoiding post hoc fitting upon completion of the search for datasets.

Our motivation and justification for the threshold choices is to reward datasets for being more open about the characteristics of the dataset and how it was collected. Open-sourced datasets can nonetheless be useful for research, irrespective of the richness of the metadata and our naming convention was chosen to recognize the current breadth of metadata available from zero upwards.

A perfect SonoDQS (1.0) score means that possible details that a majority of researchers and practitioners might want to know about a given dataset have been revealed by the publishers of the dataset. The less of those details are available, the lower the score. Those details, or characteristics, were determined in an iterative process. First, we started with the details that MICCAI Challenges^32^ are expected to include when introducing a dataset for a Challenge. Then, we cull the details that are irrelevant to ultrasound imaging specifically, while adding missing characteristics that are important to know before working on an ultrasound dataset. Then, we ranked the characteristics into two tiers to reflect their importance to a researcher who has yet to start working on a dataset.

The higher-tier characteristics have twice as much of an effect (value = 1 + 1) on the SonoDQS on the score than the lower tier (value = 1). If information pertaining characteristic is not available, a dataset gets a value = 0 for that characteristic. The values are summed together and averaged, giving us the SonoDQS score. Therefore, assuming we are only working with three characteristics where one is high-tier and is filled in, one is low-tier and is filled in, one is low-tier but not filled in, then the SonoDQS score would be ((1 + 1) + 1 + 0)/4.

Like the publicly available datasets, we also rated the open-source models. It is important to emphasize that we do not rate them based on the performance on tasks but rather how clearly the authors of the model have explained what the model does, how the model was trained, and what data it was trained on.

Inspired by RipetaScores^33^, we developed a modified scoring method tailored to open-source ultrasound models, which we call the Sonographic Model Quality Score (SonoMQS). Unlike the RipetaScore, SonoMQS omits certain criteria less relevant to this context, such as biological materials documentation. Instead, it emphasizes transparency and accessibility in deep learning models.

RipetaScores are not specific to machine learning-based models but rather a mechanism to score scientific research^33^. In a RipetaScore, there are three main considerations: reproducibility, professionalism, and research. Reproducibility focuses on whether the research being scored has enough information associated with it that would allow it to be reproduced, such as the availability of the data the model was trained on. The reproducibility category considers aspects such as the availability and location of data, the availability of code, software citations, and the documentation of biological materials. The professionalism category assesses the presence of author contribution statements, authorship clarity, competing interest declarations, ethical approval details, and funding transparency. The research category evaluates the clarity of study objectives and the presence of well-defined section headers in the manuscript. Together, these components provide a comprehensive measure of a study’s adherence to good scientific practices.

3 $${\text{RipetaScore}}=\,{\text{Research}}\,{\text{Check}}\,({\text{pass}}/{\text{fail}})\times [{\text{Professionalism}}\,(0-10)+{\text{Reproducibility}}\,(0-20)]$$

For SonoMQS, we give a model a point for affirmative (i.e., “yes”) to each of the following twelve questions: (1) training dataset is public, (2) model weights are public, (3) code is shared, (4) training details mentioned, (5) study objective mentioned, (6) funding statement mentioned, (7) ethics approval statement mentioned, (8) competing interests (conflict of interests) statement mentioned, (9) authorship mentioned, (10) authorship contribution statement mentioned, (11) code availability statement mentioned, (12) and software citations included, with questions (1), (2), (3), and (4) being weighed twice as much. The total number of points is then divided by 16 to obtain the normalized score reported in Table 2 as SonoMQS.

It is important to emphasize that SonoMQS does not measure a model’s performance in its given task. It is not a typical evaluation metric. It is a score that reflects the quality of the reporting surrounding a given model. For example, a high SonoMQS score for a model that performs segmentation does not mean that the model is excellent at segmentation, but rather that the developers of the model have been forthcoming and open about details such as how the model was trained or what the model was trained on.

## Data Availability

The collected data was made available in the tables in the main paper and the corresponding webpages. The corresponding webpages will be made available online upon paper acceptance. Since we do not train or test our own or others’ deep learning models, we consider the code availability section to not be relevant for this work.
